# Epidemiology of hepatitis C virus in Ghana: a systematic review and meta-analysis

**DOI:** 10.1186/s12879-016-1708-7

**Published:** 2016-08-09

**Authors:** Akosua Adom Agyeman, Richard Ofori-Asenso, Andy Mprah, George Ashiagbor

**Affiliations:** 1 Research Unit, Health Policy Consult, P. O. Box WJ 537, Weija-Accra, Ghana; 2 Kwame Nkrumah University of Science and Technology, Kumasi, Ghana

**Keywords:** Hepatitis C, HCV, Viral infections, Prevalence, Ghana, Systematic reviews, Meta-analysis

## Abstract

**Background:**

To fully understand the burden of hepatitis C (HCV) infection in Ghana towards informing appropriate preventive measures, accurate prevalence estimates are needed. In this study, we estimate the prevalence of chronic HCV infection by systematically reviewing primary studies published between 1995 and 2015.

**Methods:**

A systematic review and meta-analysis was conducted as per the PRISMA guidelines. Comprehensive searches for hepatitis C prevalence studies for the years 1995–2015 were conducted in PubMed, ScienceDirect, Google Scholar, Africa Journals Online (AJOL) and the WHO African Index Medicus databases. We also searched the websites of the ministry of health and Ghana Health service for non-indexed studies or reports on the subject. Further systematic reference screening of published reviews and retrieved studies were also conducted to identify additional publications not captured through the online searches.

**Results:**

Twenty-Four (24) studies from nine regions of Ghana with a combined sample size of 100,782 were analyzed. No study involving participants from Upper West region was retrieved. The national prevalence of chronic HCV was estimated as 3.0 % (95 % CI = 2.6 % to 3.5 %; I^2^ = 97.61 %, *p* < 0. 001). Prevalence rates of chronic HCV infection among blood donors was 2.6 % (95 % CI = 2.1 % to 3.1 %; I^2^ = 98.33 %, *p* < 0.001) with higher prevalence rate estimated for replacement blood donors (RBDs) than voluntary blood donors (RBDs). Among pregnant women and parturients, anti-HCV seroprevalence was estimated as 4.6 % (95 % CI = 1.8 % to 7.5 %; I^2^ = 75.74 %, *p* = 0.016). The national prevalence of HIV/HCV co-infection was also estimated as 2.8 % (95 % CI = 0.4-6 %; I^2^ = 65.86 %, *p* = 0.0053). Regional prevalence of chronic HCV infection were determined for Ashanti (1.5 %, 95 % CI = 1.2 % to 1.9 %; I^2^ = 96.24 %, *p* < 0.001) and Greater Accra (6.4 %, 95 % CI = 4.2 % to 8.6 %; I^2^ = I^2^ = 88.5 %, *P* < 0. 001) regions but no estimates were available for the other eight regions. The ascending order of HCV prevalence rates according to years in which studies were conducted was 2006–2010 < 2011–2015 < 1995–2002 < 2001–2005. Higher prevalence of chronic HCV infection was estimated for rural (5.7; 95 % CI 5.0–6.3 %; I^2^ = 0, p = 0.804) than urban (2.6 %, 95 % CI = 2.1 % to 3.0 %; I^2^ = 97.3 %, *p* = 0.0001) settings.

**Conclusion:**

Our study demonstrates a high prevalence of chronic hepatitis C infection in Ghana. This highlights the urgent need for stronger commitments from government and all stakeholders within the country to outline efficient preventive and curative measures towards reducing the overall burden of the disease.

## Background

Hepatitis C virus (HCV) infection is now considered to be of significant global health importance affecting all countries and requires the needed attention [1]. About 2.8 % of the world’s population representing almost 180 million individuals are estimated to be infected with HCV; as much as 80 % of this number suffer chronic infection—almost five times the number for HIV [2]. Although, recent estimates point to a declining burden mainly due to reduced prevalence among children, it is widely accepted that more needs to be done to control the disease [3].

Chronic HCV infection has been strongly implicated in the development of hepatocellular carcinoma (HCC). About 10–20 % of chronic HCV sufferers develop liver cirrhosis within 20–30 years of onset of infection, 1–5 % of these are likely to progress into liver cancer [4, 5]. Each year, more than 350,000 deaths from HCV-related liver diseases are recorded across the globe with majority of these deaths arising from liver cirrhosis and HCC [6]. Over 25 % of the global cases of liver cirrhosis and HCC are attributable to chronic HCV infection, with rates higher in endemic regions [7].

There are variations in the burden of HCV across the globe as depicted by prevalence rates of 1.5 %, 2.3 % and 3.2 % for the World Health Organization (WHO)’s Americas, Europe and Africa regions, respectively [1]. Madhava et al. [8] estimated the HCV prevalence in 2002 in Sub-Saharan Africa to be 3.0 % with a prevalence rate of 2.4 % for the West African region where Ghana is located. Recent estimate by Rao et al. [9] presents a slightly lower prevalence of 2.65 % for the Sub-Saharan Africa region.

However, there are concerns that prevalence rates reported for Sub-Saharan Africa may be substantially underestimated owing to factors such as the limited availability of HCV representative surveys in the region [10]. Aside the regional variations in HCV prevalence, even within countries, the patterns of HCV epidemiology vary greatly. In the United States for instance, highest HCV prevalence is recorded among persons 30-49 years, although in countries like Italy and China, persons >50 years account for most infections [11].

It is important to highlight that there are some real challenges in documenting an accurate burden of HCV to ascertain true incidence and prevalence in any country. Such challenges include for instance, the unavailability of assays with ability to distinguish acute and chronic infections as majority of acute HCV infections often present no symptoms [12].

Globally, where the impact of HCV has been thoroughly studied, the implications on national health systems has been found to be enormous [13]. Razavi et al. [14] estimated the lifetime cost of a person infected with HCV in 2011 in US to be at $64,490, although, this could rise to $205,760 ($154,890–$486,890) when medical inflation is applied. Myer et al. [15] also estimated an amount of $64,694 as the lifetime cost for Canadian with HCV infection in 2013 which could rise to as high as $327,608 if liver transplantation becomes necessary.

Viral hepatitis including HCV, are considered to be significant contributors to morbidity and mortality in Ghana and deserve greater attention [16]. However, extensive aggregate data on prevalence of HCV in Ghana are currently lacking [17]. Lavanchy [1] reported a national HCV prevalence rate of 1.7 % for Ghana in 2010 based on WHO’s data. A systematic review focusing on HCV seroprevalence in Africa by Riou et al. [18] also reported an HCV prevalence rate for Ghana within the range 0.2–9.4 %. Aside the very wide range presented by this study, the estimate was also based on only ten studies and was restricted to adult populations. To the best of our knowledge, no other thoroughly conducted systematic review and meta-analysis on the prevalence of chronic HCV infection in Ghana has been published. This observation further highlight the lack of thorough compilation of the evidence regarding the prevalence of HCV in Ghana.

To inform evidence-based policymaking, public health research and programming prioritization in Ghana, accurate prevalence estimates based on thorough and up-to-date evidence complication is essentially needed. In this paper, we aimed at estimating the prevalence of chronic HCV infection in Ghana based on studies published over the last two decades (1995–2015). This was a contribution to our large study documenting the burden of common viral hepatitis in Ghana.

## Methods

This review was conducted in accordance with recommendations outlined in the PRISMA (Preferred Reporting Items for Systematic Reviews and Meta-Analyses) statement [19].

### Search strategy

We conducted searches in PubMed, Science Direct, Google scholar, Africa Journals Online (AJOL) and the WHO African Index Medicus databases to identify entries on HCV prevalence in Ghana up to 31^st^ December 2015. The key words used were Hepatitis C, prevalence and Ghana and similar terms such as HCV and anti-HCV were crossed. The main limits used were ‘Humans’ and ‘English’. The specific search sequence for PubMed are as follows; ((“hepatitis c”[MeSH Terms] OR “hepacivirus” [MeSH Terms] OR Hepatitis C [Text Word] OR “hepatitis c antibodies”[MeSH Terms] OR anti-HCV [Text Word] OR HCV [All Fields])) AND (“prevalence” [MeSH Terms] OR prevalence [Text Word])) AND (“ghana” [MeSH Terms] OR ghana[Text Word])).

We also searched the websites of the ministry of health (http://www.moh-ghana.org/) and Ghana Health service (http://www.ghanahealthservice.org/) for non-indexed studies or reports on the subject. Cross-checking of bibliographies from other published reviews and from all retrieved articles was conducted to identify additional publications.

### Studies inclusion criteria

We included only primary studies published in English between January 1995 and December 2015 and which reported chronic HCV prevalence information among Ghanaians. This period was considered as we were primarily interested in looking at HCV prevalence over the last two decades. For a study to be included, the diagnosis of HCV should have been based on serological assays that detect antibodies to HCV; or molecular assays that aim to detect, quantify, and/or characterize HCV-RNA genomes [20]. No age restrictions were imposed and we considered studies conducted in both children and adults.

### Data extraction and quality assessment

Two reviewers (RO, AA) screened titles and abstracts against predefined study inclusion criteria. Full-text articles were also independently screened by the same reviewers (AA, RO) for eligibility. Quality appraisal of studies were carried out using a 12-point scoring system modified from the Downs and Black checklist as adopted in similar reviews [21, 22]. The criteria included for instance, whether study’s objective was clearly defined, study design clearly described, whether participants were described (e.g. blood donors, IDUs) and are representative of general population and whether ample sample size was adequate. Additionally, other measures such as whether studies accounted for missing data, cofounders discussed, and whether socioeconomic variables such as age, gender, setting (e.g. urban or rural) were fully described. Attention was also paid to whether studies reported methods by which HCV status were determined and whether the outcomes of interest were clearly spelt out and any biases reported. Studies were graded according to their scores into high, medium or low. We collected further descriptive information such as author details, study’s publication year, region of Ghana and the reported HCV prevalence. Prevalence data were independently extracted by AA and AM and crosschecked by RO. Any discrepancies were resolved through consensus-based discussions.

### Data analysis

We conducted a meta-analysis using Open Meta (analyst) software, an open-source, cross-platform software for advanced meta-analysis [23] and StatsDirect statistical software (Version 3.0.0, StatsDirect Ltd, Cheshire UK) [24]. The pooled and individual study proportions were assessed at 95 % confidence interval. We tested for heterogeneity based on Cohran’s Q statistic test and degree of inconsistency (I^*2*^) [25]. An I^2^ > 50 % was considered as representing meaningful heterogeneity. In this case, the random effect model (DerSimonian-Laird) was adopted over fixed effect model in the analysis of pooled effects [25]. We carried out direct observation of funnel plots to detect presence of publication bias and this was confirmed with Egger and Harbord statistics tests [26, 27]. A leave-one-out sensitivity analysis was carried out to assess the impact of each study on the overall pooled estimate [28]. Sub-analysis was carried out for different study periods as well as for specific populations (e.g. HIV patients, blood donors etc.) and regions of Ghana. In all computations, statistical significance was set at *p* < 0.05.

## Results

### Studies identification and characteristics

**Fig. 1:**
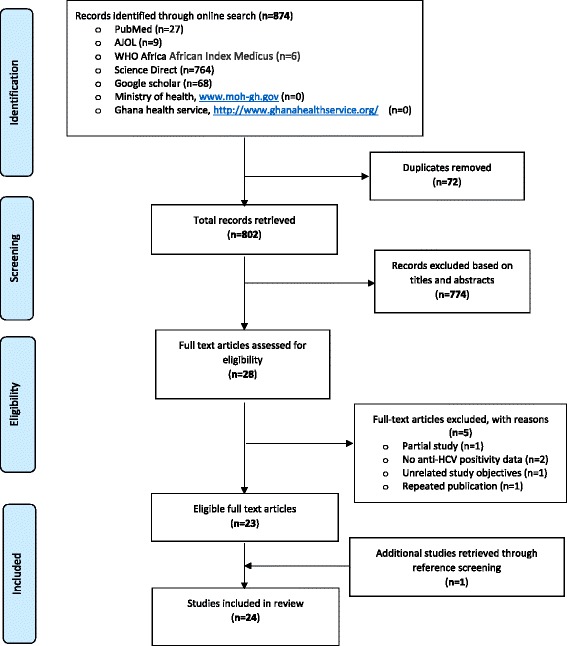
A PRISMA flow chart of studies’ retrieval steps

Figure 1 summarises the studies retrieval steps. A total of 874 citations were retrieved from the searches. Following, titles and abstract screening, removal of duplicate, and full-text analysis and referencing screening, 24 studies met the inclusion criteria for inclusion in the overall analysis [17, 29–51]. The 24 studies (Table 1) which were conducted in nine regions of Ghana altogether involved a sample size of 100,782. The regional distribution of studies were, Ashanti (12), Greater Accra (6), Northern (1), Brong-Ahafo (1), Central (1), Upper East (1) and two (2) multi-regional studies. Majority of the studies (87.5 %, *n* = 21) were conducted among urban residents. According to publication years, 25 % (*n* = 6), 33 % (*n* = 8) and 42 % (*n* = 10) of studies were published within the periods 1995–2002, 2003–2009 and 2010–2015 respectively. Sample sizes across the 24 studies ranged from 110 to 51,100. A significant proportion (54 %, *n* = 13) of the studies were conducted among blood donors (either replacement or voluntary donors) participants. Only one study was specifically conducted in children [44]. Three studies were conducted among specific disease group of patients (diabetes and HIV). The quality appraisal scores graded 4 %, 38 % and 58 %, of studies to be of low, medium and high quality, respectively.

**Table 1:** Descriptive characteristics of studies reporting chronic HCV prevalence in Ghana

Study No	Author details	Year of publication	Design	Region of study	Study population	Age group (yrs.)	Setting	Sample size (n)	Method	Anti-HCV (%)	Quality grade
1	Acquaye and Tetteh-donkor. [29]	2000	Cross-sectional	Greater Accra	Blood donors^€^	n.s	Urban	1300	ELISA	5.2	Medium
2	Adoba et al. [30]	2015	Cross-sectional	Ashanti	Barbers	28.18^a^	urban	200	Rapid test	0.5	High
3	Adjei et al. [31]	2006	Cross-sectional	Eastern & Greater Accra	Prison inmates & Officers	17–84	Urban	363	ELISA	20.1	High
4	Adjei et al. [32]	2008	Cross-sectional	National (Excludes Upper East & West)	Prison Officers	38.1^b^	Urban	445	ELISA	18.7	High
5	Allain et al. [33]	2009	Cross-sectional	Ashanti	Blood donors^€^	>16	Urban	51100	Rapid test	0.4	High
6	Allain et al. [34]	2010	Cross-sectional	Ashanti	Blood donors^€^	31.0^a^	Urban	11000	Rapid test	0.22	High
7	Amidu et al. [35]	2010	Cross-sectional	Upper East	Blood donors^€^	17-58	Urban	4146	Rapid test	3.6	Medium
8	Ampofo et al. [17]	2002	Cross-sectional	Greater Accra	Blood donors^€^	16-60	Urban	808	Agglutination assay	8.4	High
9	Apea-Kubi et al. [36]	2006	Cross-sectional	Greater Accra	Pregnant & non-pregnant women	29.6^a^	Urban	517	Rapid test	5.2	High
10	Blankson et al. [37]	2005	Case-control	Greater Accra	Cirrhotic & non-cirrhotic patients	15–90	Urban	350	ELISA	4.3	High
11	Candotti et al. [38]	2001	Cross-sectional	Ashanti	Blood donors^€^	32^a^	Urban	2738	EIA	1.3	High
12	Candotti et al. [39]	2003	Cross-sectional	Ashanti	Blood donors^€^	29^a^	Urban	4984	EIA	1.3	Medium
13	Ephraim et al. [40]	2014	Cross-sectional	Central	Diabetics	n.s	Urban	110	Rapid test	0.0	Medium
14	Ephraim et al. [41]	2015	Cross-sectional	Ashanti	Pregnant women	10–40	Urban	168	Rapid test	7.7	Medium
15	King et al. [42]	2014	Cross-sectional	Ashanti	HIV+ individuals	n.s	Urban	408	Plasma assay	1.0	High
16	Kubio et al. [43]	2012	Register Review	Northern	Blood donors^€^	n.s	Rural	819	n.s	6.1	Low
17	Lassey et al. [44]	2004	Cross-sectional	Greater Accra	Parturients	n.s	Urban	638	ELISA	2.5	Medium
18	Martinson et al. [45]	1996	Cross-sectional	Ashanti	Children	6–18	rural	803	n.s	5.4	Medium
19	Nkrumah et al. [46]	2011	Cross-sectional	Ashanti	Blood donors^€^	26–35	Rural	2773	Rapid test	5.63	Medium
20	Owusu-Ofori et al. [47]	2005	Cross-sectional	Ashanti	Blood donors^€^	n.s	Urban	9372	Rapid test	0.5	High
21	Sagoe et al. [48]	2012	Cross-sectional	Greater Accra	HIV+ individuals	≥18	Urban	138	Plasma assay	3.6	High
22	Sarkodie et al. [49]	2001	Cross-sectional	Ashanti	Blood donors^€^	16–52	Urban	3264	Mixed methods	1.6	High
23	Walana et al. [50]	2014	Cross-sectional	Brong-Ahafo	Blood donors^€^	20–49	Urban	3402	ICT	4.4	Medium
24	Wansbrough-Jones et al. [51]	1998	Cross-sectional	Ashanti	Pregnant women & blood donors^€^	15–60	Urban	936	ELISA	2.8	High

*n.s* not specified, *ICT* immunochromatography, *ELISA* enzyme-linked immunosorbent assay, *EIA* enzyme immunoassays, *anti-HCV* hepatitis C antibody

^a^average

^b^median

^€^Blood donors is used to represent either voluntary or replacement donors or both

### Overall national prevalence

**Fig. 2:**
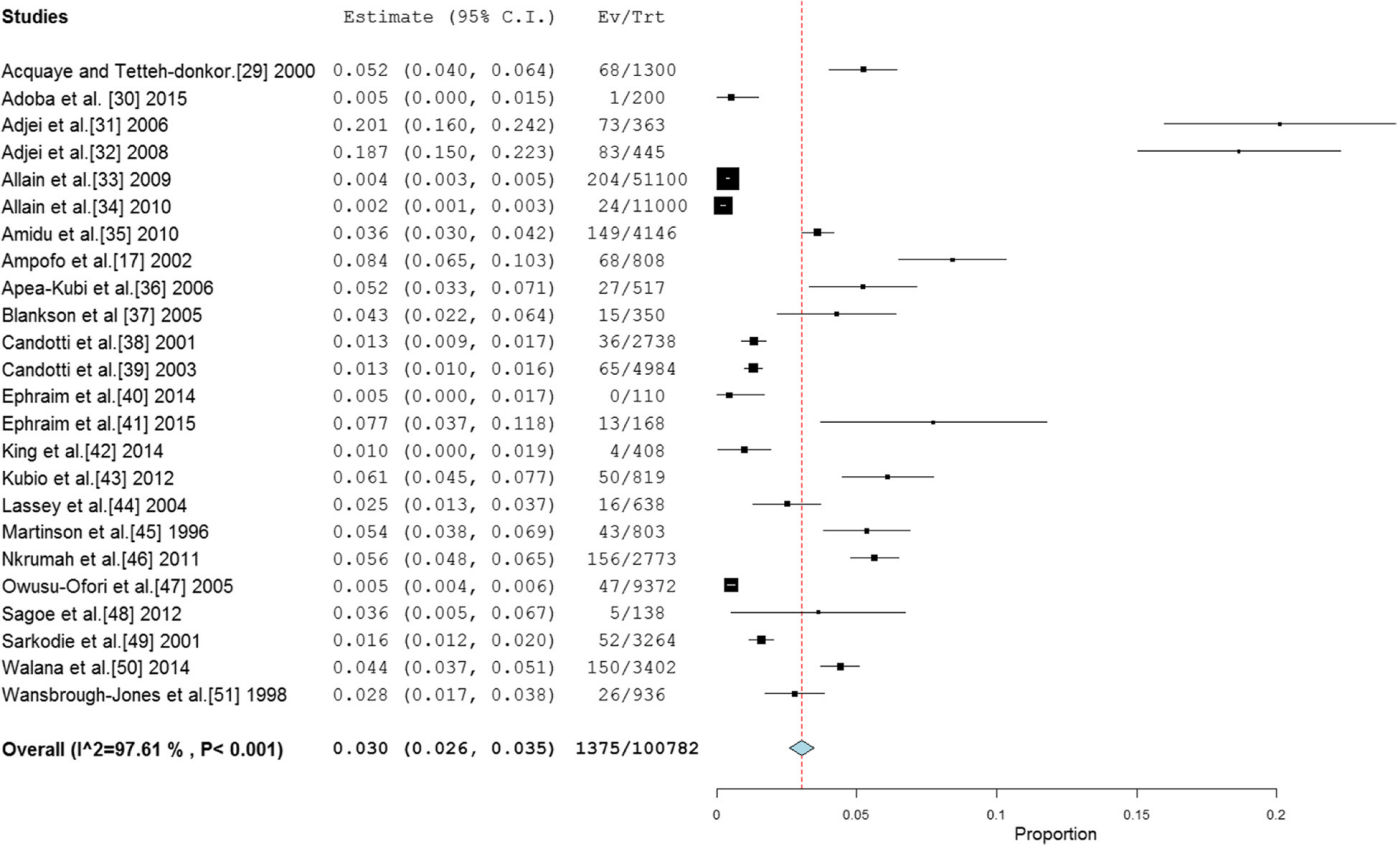
Forest plot of studies reporting chronic HCV infection prevalence in Ghana

The reported anti-HCV prevalence rate across the 24 studies ranged from 0 % to 20.1 %. In 63 % (15/24) of studies, the reported prevalence was more than 2 %— the level at which anti-HCV prevalence rate is considered to be high [52]. 42 % of studies reported prevalence rates at least 200 % higher than the 2 % level. The pooled national prevalence estimate (Fig. 2) was determined as 3.0 % (95 % CI = 2.6 % to 3.5 %). The result of heterogeneity (*I*^*2*^) was also 97.61 % (*p* < 0.001) for the degree of inconsistency.

### Blood donors

**Fig. 3:**
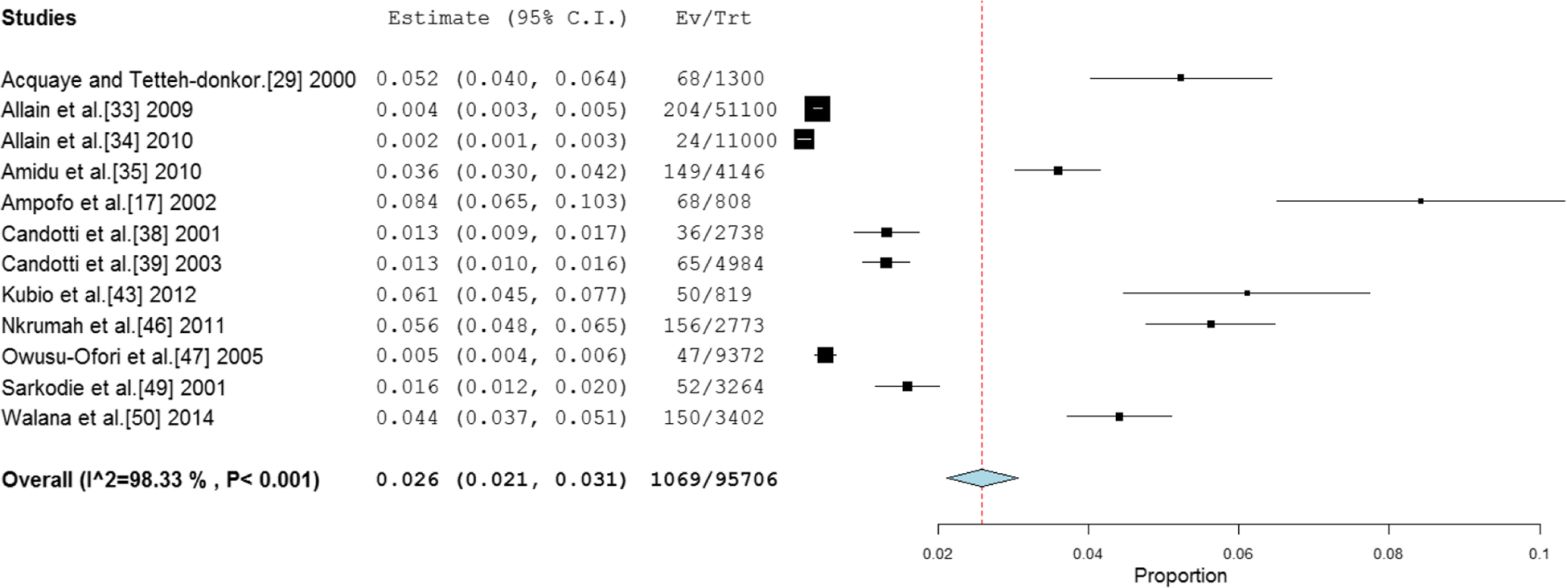
Forest plot of studies reporting chronic HCV prevalence among blood donors in Ghana

A total of thirteen (13) studies involved blood donor participants (voluntary and family replacement donors), although data on anti-HCV prevalence for this specific population obtained from twelve (12) studies. The 12 studies altogether involved a sample size of 95,706. The reported HCV prevalence was in the range of 0.22 to 8.4 %. The pooled estimate of HCV prevalence among blood donors (Fig. 3) across the twelve (12) studies was 2.6 % (95 % CI = 2.1 % to 3.1 %). The result of heterogeneity (*I*^*2*^) was also 98.33 % (*p* < 0.001) for the degree of inconsistency. Separate prevalence for VBDs and RBDs were retrieved from five studies [34, 38, 43, 47, 49]. The pooled prevalence of HCV prevalence rate for VBDs was determined as 0.3 % (95 % CI 0.1–0.5 %). The result of heterogeneity (*I*^*2*^) was also 69.04 % (*p* < 0.0001) for the degree of inconsistency. On the other hand, the pooled HCV prevalence for RBDs was determined as 1.8 % (95 % CI 0.9–2.6 %). The result of heterogeneity (*I*^*2*^) was determined as 95.42 % (*p* < 0.001). The prevalence difference of 1.5 % (95 % CI 1.3–1.8 %) between RBDs and VBDs was found to be statistically significant (*p* < 0.0001).

### Pregnant women and parturients

**Fig. 4:**
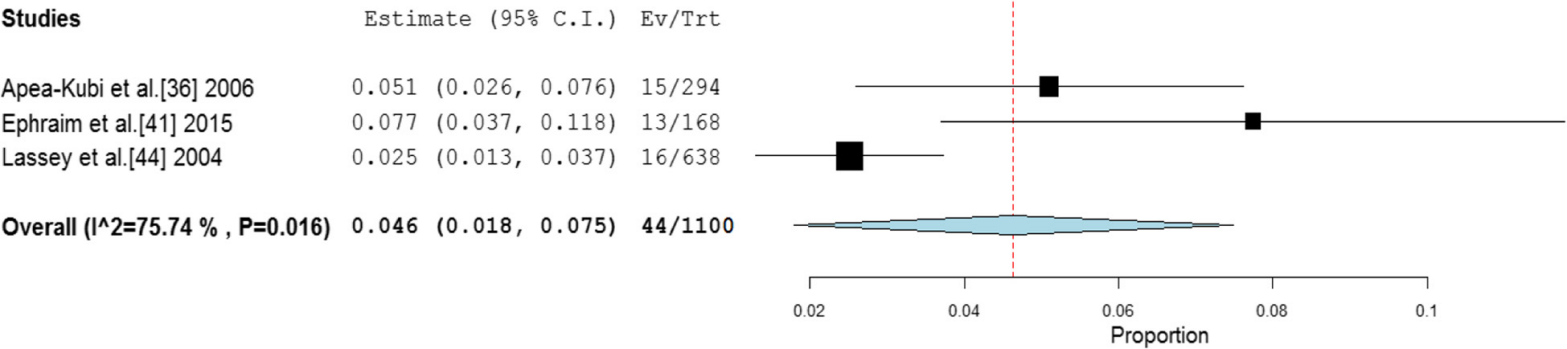
Forest plot of studies reporting chronic HCV prevalence amongst pregnant women and parturients in Ghana

Four studies involved pregnant women and parturient participants [36, 41, 44, 51]. One of these studies [51], did not present any specific prevalence data on pregnant women and thus was excluded from this analysis. Hence anti-HCV prevalence among pregnant women and parturients were retrieved from three studies which together involved a total of 1,100 participants. The HCV prevalence across these studies was within the range 2.5 to 7.7 %. The pooled HCV prevalence estimate for the pregnant women and parturients was determined as 4.6 % (95 % CI = 1.8 % to 7.5 %) (Fig. 4). The result of heterogeneity (*I*^*2*^) was determined as 75.74 % (*p* = 0.016) for the degree of inconsistency.

### High risk groups

Persons at greater risk of chronic HCV include injection drug users (IDUs), HIV patients, people in custodial settings and commercial sex workers [53–55]. Two studies [31, 32] were conducted in prisons although data among prisoners was retrieved from only one study [31]. An extremely high (19.2 %) prevalence of HCV was recorded among the incarcerated persons. Reported illegal drug use was high among this group (83.2 % reporting using marijuana, 7.3 % reported cocaine use, 5.2 % indicate history of heroin abuse and 4.2 % used phencyclidine) [31]. Prevalence of HCV among HIV positive individuals was retrieved from three studies [42, 43, 48]. The pooled prevalence of chronic HCV among HIV patients from the three studies was determined as 2.8 % (95 % CI = 0.4–6 %). The result of heterogeneity (*I*^*2*^) was determined as 65.86 % (*p* = 0.0053) for the degree of inconsistency. No study reported individual prevalence rate among IUDs as well as for commercial sex workers.

### HCV prevalence for rural and urban settings

**Fig. 5:**
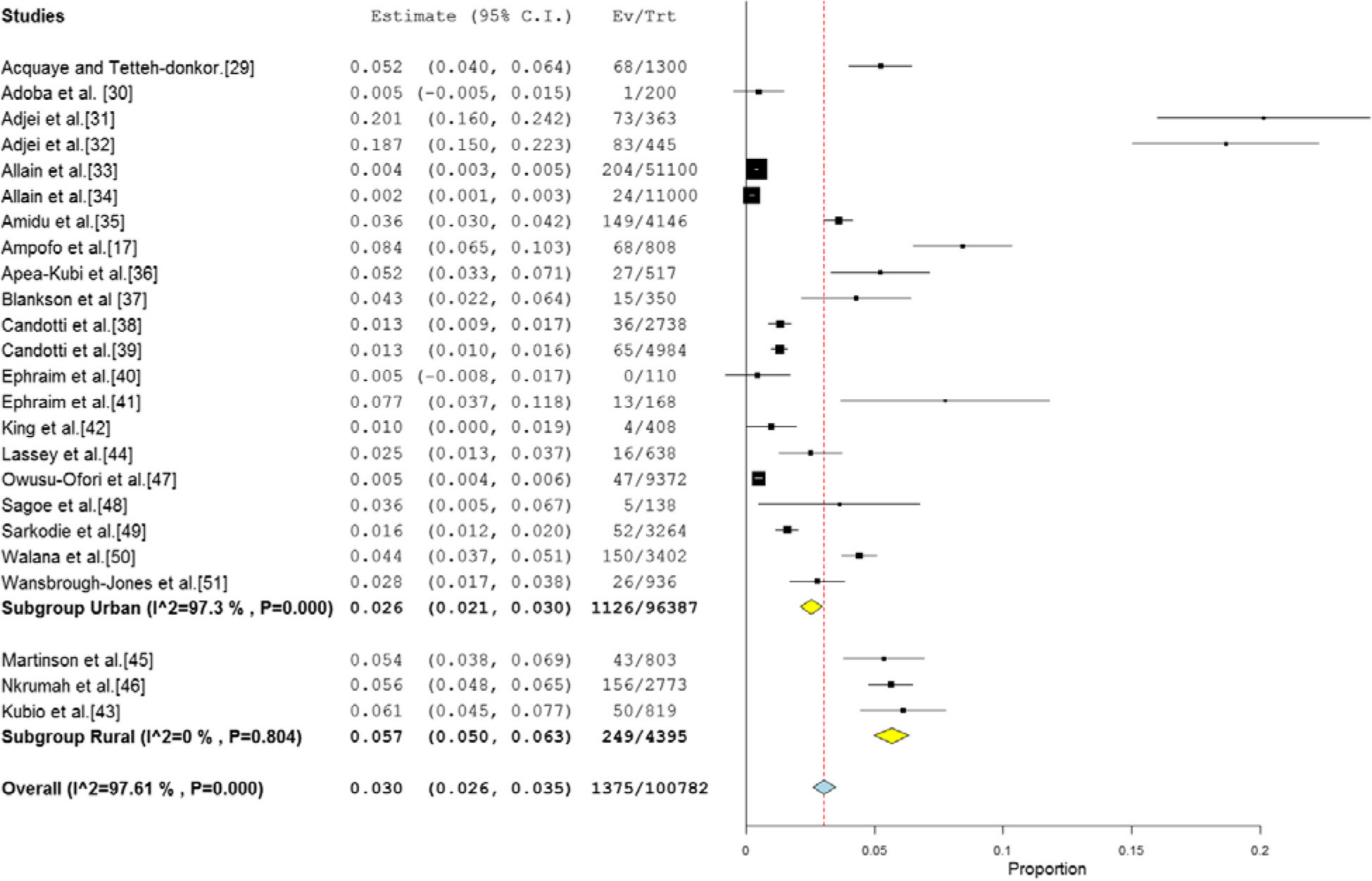
Forest plot of HCV infection for studies conducted in urban and rural parts of Ghana

Three (3) studies were conducted among rural dwellers [43, 45, 46]. These studies involved a total of 4,395 participants and reported individual HCV prevalence ranging from 5.4 to 6.1 %. The pooled HCV prevalence estimate for urban setting was determined as 5.7 % (95 % CI 5.0–6.3 %; I^2^ = 0; *p* = 0.804). Twenty-one (21) studies involved urban dwellers and together included a total of 96,387 participants. The reported individual HCV prevalence ranged from 0 to 20.1 %. The pooled prevalence estimate (Fig. 5) for the studies conducted in urban settings was 2.6 % (95 % CI = 2.1 % to 3.0 %). The result of heterogeneity (*I*^*2*^) was determined as 97.3 % (*p* = 0.001) for the degree of inconsistency. The difference in pooled HCV prevalence estimates for rural and urban settings was statistically significant (*p* < 0.002).

### Regional prevalence

**Fig. 6:**
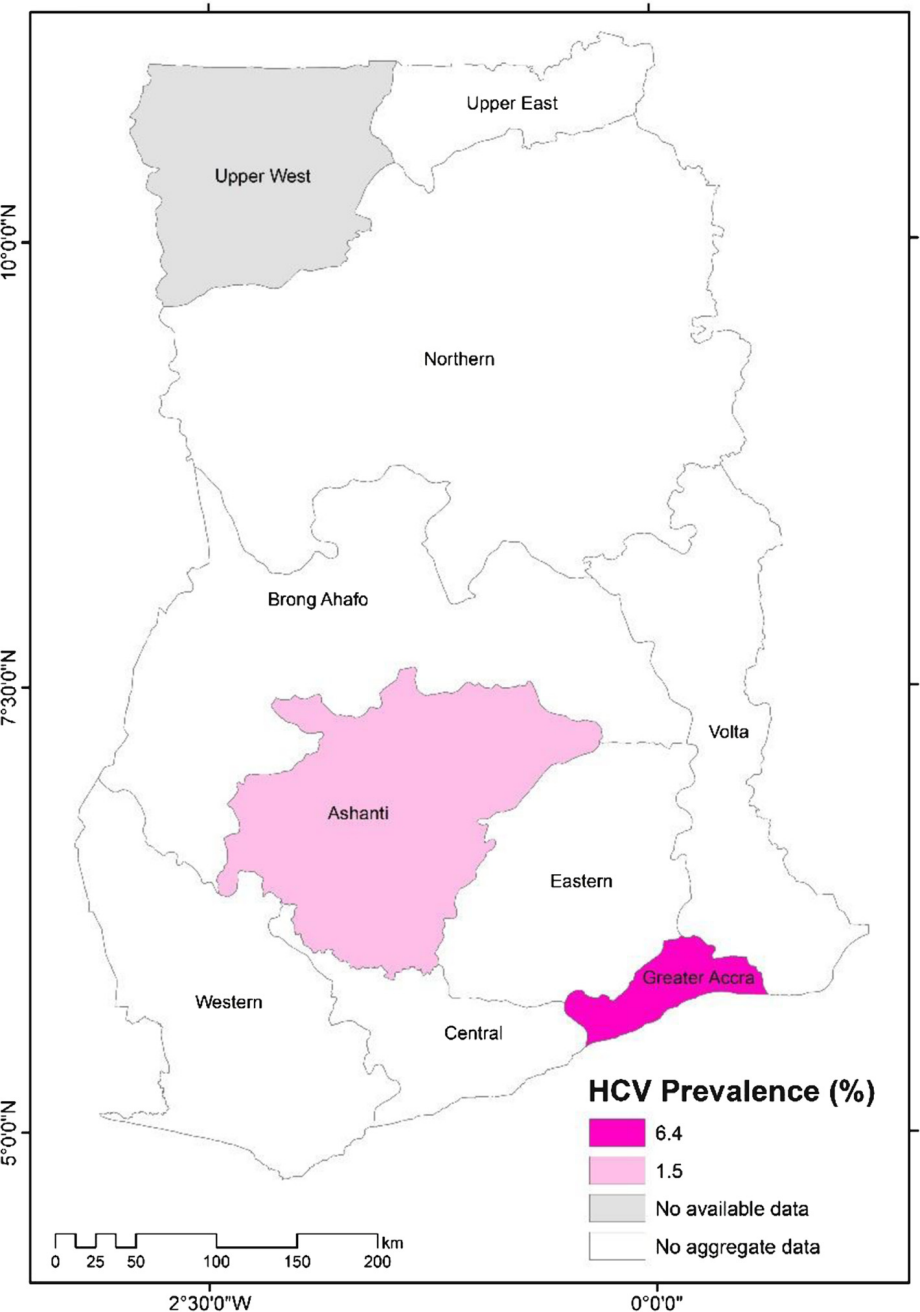
A map of chronic HCV prevalence across regions of Ghana

Pooled HCV prevalence estimate for the Ashanti region was estimated from 13 studies which altogether involved 87,805 participants. The reported HCV prevalence rates across the thirteen (13) studies ranged from 0.22 to 11.9 %. The pooled HCV prevalence estimate for Ashanti region was determined as 1.5 % (95 % CI = 1.2 to 1.9 %; I^2^ = 96.24; *p* < 0.001). Pooled HCV prevalence for the Greater-Accra Region, was estimated from eight studies which altogether involved 4,063 participants. The reported individual HCV prevalence from the eight studies ranged from 2.5 to 17.8 %. The pooled HCV prevalence for the Greater-Accra region was determined as 6.4 % (95 % CI = 4.2 % to 8.6; I^2^ = 88.5 %; *p* < 0.001). We were unable to deduce aggregate data for Eastern, Upper East, Brong-Ahafo, Central, Northern, Western and Volta regions due to limited number of studies and no single reported data was available for Upper West region (Fig. 6).

### HCV prevalence according to periods in which studies were conducted

**Fig. 7:**
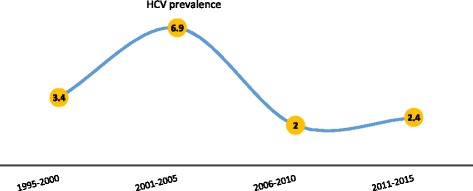
HCV infection prevalence according to periods in which studies were conducted

Studies were grouped according to the mid-year of the period in which they were undertaken. The four period groupings were 1995–2000, 2001–2005, 2006–2010 and 2011–2015. Among the seven studies conducted within the period 1995–2000 which involved a total 14,833 participants, the reported individual HCV prevalence ranged from 1.3 to 8.4 %. The pooled HCV prevalence estimates for the studies conducted in the period 1995–2000 was 3.4 % (95 % CI 2.3 to 4.4 %; I^2^ = 94.76; *p* < 0.001). The seven studies conducted within the period 2001–2005 also involved a sample population of 15,831 and reported HCV prevalence within the range of 0.5–20.5 %. The pooled HCV prevalence estimate for the studies conducted in the period 2001–2005 was determined as 6.9 % (95 % CI 4.4 to 9.4 %; I^2^ = 98.16; *p* < 0.001). Five studies were conducted in the period 2006–2010 and together involved 65,830 participants and reported HCV prevalence ranging from 0.2 to 6.1 % with a pooled HCV prevalence estimate of 2.0 % (95 % CI 1.4–2.6 %; I^2^ = 98.08; *p* < 0.001). Among the five studies conducted within the period 2011–2015 which altogether involved a total of 14,833 participants, the reported HCV prevalence ranged from 1.3 to 8.4 %. The pooled estimate for the studies conducted in the period 2011–2015 was 2.4 % (95 % CI =0.3 to 4.5 %; I^2^ = 94.42; *p* < 0.001). Hence the order of ascending HCV infection prevalence rates according to years in which studies were conducted was 2006–2010 < 2011–2015 < 1995–2002 < 2001–2005 (Fig. 7).

### Heterogeneity assessment

**Fig. 8:**
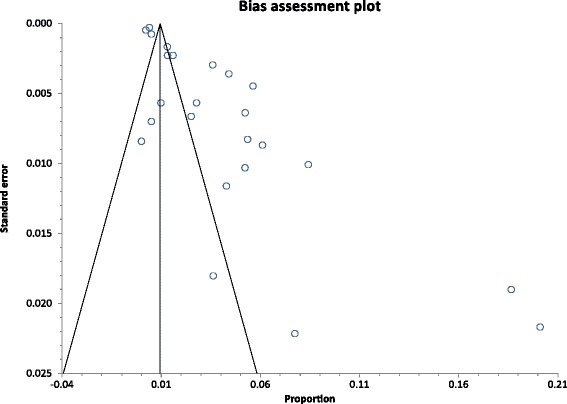
A funnel plot of studies reporting chronic HCV infection prevalence in Ghana

**Fig. 9:**
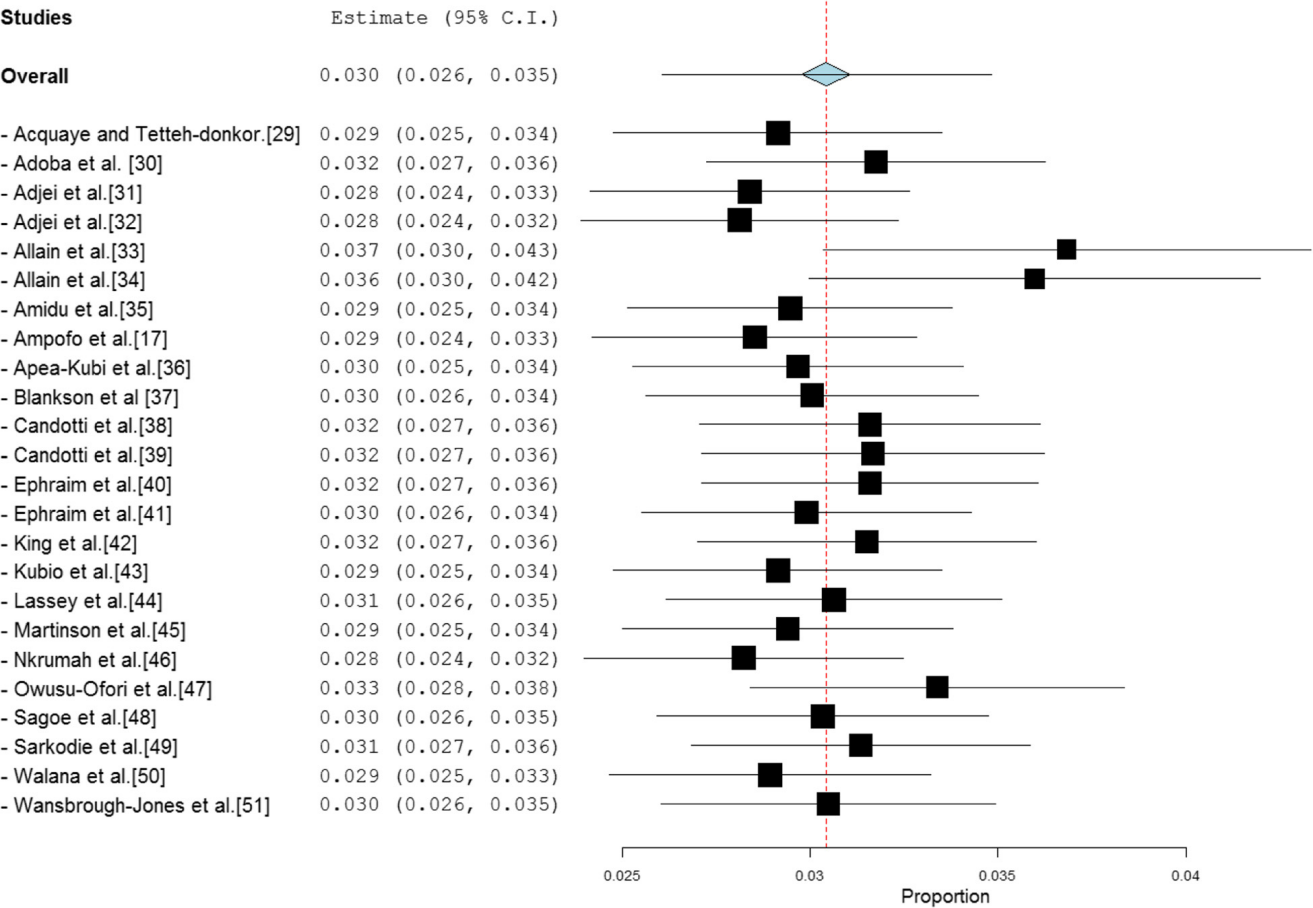
A leave-one-out sensitivity plot of studies reporting chronic HCV infection prevalence in Ghana

A direct observation of funnel plot revealed an asymmetrical display of the HCV prevalence reported by the various studies (Fig. 8). The presence of publication bias was confirmed by statistically significant Egger (*p* < 0.0001) and Harbord (*P* = 0.0002) tests. A leave-one-out analysis which was performed to assess the impact of the various studies on the pooled estimates [32], showed that the pooled HCV prevalence estimate was dominated by Allain et al. [37] and Allain et al. [38]. The most dominant studies all involved blood donors (Fig. 9).

## Discussion

In this review, we present a high prevalence (3.0 %) of chronic HCV infection in Ghana. This prevalence rate is significantly higher than the national prevalence of 1.7 % reported previously by Lavanchy [1]. The observation of higher prevalence rate in this study may be in alignment with the observation by Layden et al. [10] that HCV prevalence rates for sub-Saharan Africa have generally been underestimated in the past. Even that, the prevalence rate reported may still be modest considering that a significant proportion of studies included in this review involved low risk populations such as blood donors who are often biased towards a healthier population [56]. Additionally, as highlighted in the sensitivity analysis, the anti-HCV prevalence was most impacted by two studies all conducted in blood donors [37, 38]. In developed countries like Australia and the US, prevalence of chronic HCV infection has been estimated to be less than 2 % [7]. This may highlight that the level of chronic HCV infection in Ghana may be considerably high.

The high prevalence of chronic HCV infection together with previously reported high prevalence of other viral hepatitis in Ghana [57], points to a growing concern regarding the safety of blood products in the country. Although, Ghana has a national blood policy which requires that all donated blood are screened for blood-borne infections including HIV 1 and 2, Hepatitis B (HBV), HCV and Syphilis [58], a greater emphasis and stricter monitoring to ensure high levels of adherence to this policy will be needed to minimise the risk of supplying contaminated blood to patients.

The prevalence of chronic HCV infection among pregnant women was estimated as 4.6 % which is high when reference is made to other regions like the United States and Europe where prevalence of chronic HCV among this group has been estimated to be around 1–2.5 % [59]. Our result therefore highlight the importance of vertical transmission in the overall spread of HCV infection in Ghana. Studies have demonstrated that chronic HCV infection during pregnancy poses significant risks to mother and child and may further exacerbate the risks of preterm delivery, low birth weight, congenital malformations, glucose intolerance during pregnancy and overall perinatal mortality [60, 61]. The high HCV prevalence among pregnant women draws attention to the need for the adoption of a national program that includes HCV screening for all or most-at-risk pregnant women in Ghana. Although, a vaccine to prevent HCV infection is currently non-existent, treatment with preparations such as interferon for women with high HCV viral load will lead to reduction in HCV infection levels for the women and also minimise the risks of vertical transmission of the disease in future pregnancies [59].

Several factors may contribute to the observed high levels of chronic HCV infection in Ghana. Studies have reported low levels of awareness and knowledge among Ghanaians about the transmission pathways of the disease. For instance, Mutocheluh and Kwarteng [62], reported that among 200 barbers in Kumasi none (0 %) of the barbers could describe the common HCV transmission pathways and only 7 % were aware that sharing razor blade or hair trimmer could be a means of transmitting viral pathogens like HCV and HBV. Similar trends of low awareness has been reported for pregnant women [63]. Additionally, while the transmission dynamics of HCV remains varied and include unprotected sex, mother-to-child transmission and transfusion of infected blood, in many Ghanaian communities, there has been an over-emphasis on HCV and related viral hepatitis as sexually transmitted infections (STIs) [13]. Such perceptions have often led to HCV-positive individuals been stigmatized and at times discouraged such patients seeking proper care to minimise their chances of spreading the disease.

Controlling HCV will require three (3) key strategies which include offering treatment for infected individuals, implementing measures to halt the transmission of the disease and reducing mortality arising from unmanaged complications such as HCC [7]. Antiviral medications have shown great potency in curing HCV infections (approximately 90 % of infections) but access to these medicines remain poor in many developing countries including Ghana [64]. To ensure that such therapies are available to majority of chronic HCV sufferers in Ghana, they must be provided freely under the country’s National Health Insurance Scheme (NHIS). This must be one of the key focus or priority for the government considering that wider availability of highly effective new generation direct-acting antiviral medications to treat HCV infections would not only reduce the occurrence of complications such as HCC but will also lead to decline in prevalence of the disease arising from altered infectivity state of treated individuals.

Unfortunately, unlike HBV, a vaccine for HCV is currently unavailable and therefore interruption of infection transmission through risk reduction would rely mainly on education to improve knowledge and awareness of the transmission dynamics of the disease. While routine screening of the general population may not be recommended as it’s deemed to be not cost effective, screening of high-risk groups such as injection drug users (IDUs) and persons with high-risk sexual behaviours should be expanded nationwide and provided possibly freely to increase patronage. This should be backed with other innovative strategies such as provision of comprehensive harm-reduction services to injection drug users including sterile injecting equipment [64]. Additionally, attention needs to be paid to conditions in the prisons as the risk of HCV infection remains high in these settings. Agyei et al. [38], reported that as much as 35.2 % of prisoners reported ever injecting drugs and of these 11.5 % were HIV-positive. Public health interventions should aim at addressing the specific needs of such populations as they may pose additional transmission risk once released into the community [37, 38].

Horizontal transmission of HCV has been recognized to be linked to factors such as age, socioeconomic/living conditions as well as other risky behaviours such as sharing towels, dental cleaning materials and chewing gum [45]. Martinson et al. [45], for instance, has demonstrated that general improvements in socio-economic conditions may lead to a decreased exposure to viral hepatitis including hepatitis B and HCV among Ghanaians. Over the last few decades, there have been significant improvement in the living conditions of Ghanaians. For instance, the percentage of Ghanaians with improved access to water increased from 56.0 % in 1990 to 86.0 % in 2012 [65]. The proportion of Ghanaians classified as poor has also decreased by more than 50 % from 52.6 % in 1991 to 21.4 % in 2012 [66]. Although, studies conducted after 2005 generally reported lower HCV prevalence than those conducted before, in the context of limited data it is difficult to assess the extent to which general improvements in socioeconomic conditions have contributed to reductions in the HCV burden across the country and for different regions. The high prevalence of HCV in Greater Accra may be due to the region’s national status of harbouring the capital city. This has often resulted in a high influx of both young and old into its commercial areas from all corners of the country and internationally for business, tourism or to seek greener pastures, an atmosphere which provides a conducive environment for the promotion of anti-social behaviours such as drug abuse and prostitution; patterns which may facilitate HCV transmission [67].

Although, limited studies have been conducted to evaluate the impact of chronic HCV infection on Ghana’s health system and economy in general, the impact arising from the high prevalence of the disease may be enormous owing to significant mortality and costs associated with complications such as HCC and liver cirrhosis. The contribution of HCV to liver cirrhosis was demonstrated in a study by Blankson et al. [37], which identified that chronic HCV infection was implicated in 1 in 14 liver cirrhosis cases. Treatment for chronic HCV is deemed to be expensive. A standard 12-week treatment with one of the newer drugs Sovaldi® (Sofosbuvir) cost around US $ 84,000 which breaks down to $1000 for each pill taken daily [68]. This is certainly a cost that most HCV Ghanaians cannot afford neither could it be absorbed by any sustainable public funding. While generic versions of such medications cost significantly less, there remains major availability issues. The high prevalence of HCV infection in HIV patients is also likely to increase mortality in such groups. While majority of studies did not document the most prevalent age groups, high HCV burden is likely to impact on economic growth as productivity among affected persons are likely to be affected and their general wellbeing diminished. Thus, high HCV burden can have significant impact on Ghana’s economy and as such a strong economic argument for government commitment and intervention in tackling the disease should be made.

Reducing the overall burden of chronic HCV infection in Ghana will require new measures and strategies and the recognition of the disease as one of the key country’s priority areas. Recently, the World Health Organization (WHO) introduced updated guidelines as a framework for countries to plan the expansion of clinical services to persons suffering chronic HCV infection [69]. The guideline highlights nine (9) key areas such as screening of high-risk, groups, mitigating liver damage through measures such as alcohol assessment and treatment with appropriate regimen such as interferon [69]. The government of Ghana would need to pay closer attention to implementing the recommendations set out in this guideline if the battle against HCV is to be won. Tackling the burden of chronic HCV infection in Ghana would also require stronger commitments from government and all other interest groups with the objective of ensuring that measures to control HCV are fully incorporated in national policies. The role of civil societies and pressure groups in driving such changes have been widely recognized and their involvement in for instance, the fight against the HIV/AIDS epidemic has been referenced as a remarkable achievement including successfully pushing for the reduction in the cost of ARTs. The successes and experience from HIV/AIDS epidemic should guide future strategies against viral hepatitis such as HCV [70].

### Strengths and limitations

The screening method employed can significantly impact on the HCV prevalence rate [71]. Since our review covered a two decade period, the studies adopted different screening techniques there are likely vary in terms of sensitivity and specificity which could partly account for the difference in prevalence rates across studies published in different years. As indicated by Fox [72], it can be extremely difficult to differentiate acute from chronic HCV infection especially in patients who have not previously undergone anti-HCV testing and it is possible that the prevalence rate reported in some studies may not be representative of only chronic infections. While this review highlights a growing evidence in this area of research as depicted by almost half (48 %) of studies were recently published (within last 5 years), there are significant regional variations. Nearly two-thirds (75 %) of studies as well as around 90 % of total population size involved were from two regions (Ashanti and Greater Accra), although these two regions represented just a little over 35 % of the country’s population in 2010 [73]. Also it was not possible to deduce aggregate HCV prevalence estimates for seven regions and no data was retrieved from the upper west region. A wider epidemiological study conducted around the same time may help to provide a more accurate prevalence estimate as well as minimize the wide heterogeneity as observed in the studies reviewed. Additionally, most studies involved adult participants with only one study specifically conducted in children [45]. Likewise, most studies reported no information on HCV prevalence in relation to demographic (e.g. age and sex) and socio-economic (e.g. education) as well as risk profiles (e.g. blood transfusion, multiple injection drug use, HIV status) which are all known predictors of HCV status [74]. It will be important for future research to look into the epidemiology of HCV in the underrepresented regions as well as targeted high-risk groups to provide greater insight into the HCV burden in Ghana. In spite of the above limitations, this review presents a more rigorous estimate of chronic HCV infection in Ghana that should inform health planning, policy decisions and the design of public health strategies by the ministry of Health and the Ghana Health service towards controlling the disease.

## Conclusion

This study has documented a high prevalence of chronic HCV infection in Ghana. The results highlight the need for urgent public health interventions aimed at reducing the infection rate. These must include efforts to increase awareness and knowledge about HCV transmission dynamics, targeting the screening of high-risk groups and providing treatment for affected individuals. Further research is also needed to understand fully the population factors underlying this high HCV prevalence. New studies particularly in underrepresented regions and high-risk population groups are urgently needed to offer better perspective on the overall HCV burden in Ghana.

## Abbreviations

HCV, hepatitis C virus; WHO, World Health Organization; HBV, hepatitis B virus; Anti-HCV, hepatitis C antibody; NHIS, National health insurance scheme; HDI, human development index; HIV, human immunodeficiency virus; STI, sexually transmitted infection

## References

[1] Lavanchy D (2011). Evolving epidemiology of hepatitis C virus. Clin Microbial Infect.

[2] Messina JP, Humphreys I, Flaxman A, Brown A, Cooke GS, Pybus OG, Barnes E (2015). Global distribution and prevalence of hepatitis C virus genotypes. Hepatology.

[3] Gower E, Estes C, Blach S, Razavi-Shearer K, Razavi H (2014). Global epidemiology and genotype distribution of the hepatitis C virus infection. J Hepatol.

[4] The Global burden of Hepatitis C working Group (2004). Global burden of disease (GBD) for hepatitis C. J Clin Pharmacol.

[5] Di Bisceglie AM, Order SE, Klein JL (1991). The role of chronic viral hepatitis in hepatocellular carcinoma in the United States. Am J Gastroenterol.

[6] Perz JF, Armstrong GL, Farrington LA, Hutin YJ, Bell BP (2006). The contributions of hepatitis B virus and hepatitis C virus infections to cirrhosis and primary liver cancer worldwide. J Hepatol.

[7] Averhoff FM, Glass N, Holtzman D (2012). Global burden of hepatitis C: considerations for healthcare providers in the United States. Clin Infect Dis.

[8] Madhava V, Burgess C, Drucker E (2002). Epidemiology of chronic hepatitis C virus infection in sub-Saharan Africa. Lancet Infect Dis.

[9] Rao VB, Johari N, du Cros P, Messina J, Ford N, Cooke GS (2015). Hepatitis C seroprevalence and HIV co-infection in sub-Saharan Africa: a systematic review and meta-analysis. Lancet Infect Dis.

[10] Layden JE, Phillips R, Opare-Sem O, Akere A, Luke A, Tayo BO, Cooper RS (2014). Hepatitis C in sub-Saharan Africa: urgent need for attention. Open Forum Infect Dis.

[11] Alter MJ (2007). Epidemiology of hepatitis C infection. World J Gastroenterol.

[12] Mohd Hanafiah K, Groeger J, Flaxman AD, Wiersma ST (2013). Global epidemiology of hepatitis C virus infection: new estimates of age-specific antibody to HCV seroprevalence. Hepatology.

[13] Adler M, Goubau P, Nevens F, Van Vlierberghe H (2002). Hepatitis C virus: the burden of the disease. Acta Gastroenterol Belg.

[14] Razavi H, ElKhoury AC, Elbasha E, Estes C, Pasini K, Poynard T, Kumar R (2013). Chronic Hepatitis C Virus (HCV) disease burden and cost in the United States. Hepatology.

[15] Myers RP, Krajden M, Bilodeau M, Kaita K, Marotta P, Peltekian K, Ramji A, Estes C, Razavi H, Sherman M (2014). Burden of disease and cost of chronic hepatitis C virus infection in Canada. Can J Gastroenterol Hepatol.

[16] Hepatitis Foundation of Ghana. Viral Hepatitis in Ghana: The Role of the Government. 2014. Available online at http://theobaldhepb.org/?page_id=624. Accessed 01 Jan 2016.

[17] Ampofo W, Nii-Trebi N, Ansah J, Abe K, Naito H, Aidoo S, Nuvor V, Brandful J, Yamamoto N, Ofori-Adjei D, Ishikawa K (2002). Prevalence of blood-borne infectious diseases in blood donors in Ghana. J Clin Microbiol.

[18] Riou J, Aït Ahmed M, Blake A, Vozlinsky S, Brichler S, Eholié S, Boëlle PY, Fontanet A (2016). HCV epidemiology in Africa group. Hepatitis C virus seroprevalence in adults in Africa: a systematic review and meta-analysis. J Viral Hepat.

[19] Moher D, Liberati A, Tetzlaff J, Altman DG (2009). Preferred reporting items for systematic reviews and meta-analyses: the PRISMA statement. Ann Intern Med.

[20] Gretch DR (1997). Diagnostic tests for hepatitis C. Hepatology.

[21] Musa B, Bussell S, Borodo MM, Samaila AA, Femi OL (2015). Prevalence of hepatitis B virus infection in Nigeria, 2000–2013: A systematic review and meta-analysis. Niger J Clin Pract.

[22] Downs SH, Black N (1998). The feasibility of creating a checklist for the assessment of the methodological quality both of randomized and non-randomized studies of health care interventions. J Epidemiol Community Health.

[23] Wallace BC, Dahabreh IJ, Trikalinos TA, Lau J, Trow P, Schmid CH (2012). Closing the gap between methodologists and end-users: R as a computational back-end. J Stat Softw.

[24] StatsDirect. Proportion Meta-analysis http://www.statsdirect.com/help/default.htm#meta_analysis/proportion.htm. Accessed 04 Feb 2016.

[25] Higgins JPT, Thompson SG, Deeks JJ, Altman DG (2003). Measuring inconsistency in meta-analyses. Br Med J.

[26] Egger M, Davey Smith G, Schneider M, Minder C (1997). Bias in meta-analysis detected by a simple, graphical test. BMJ.

[27] Harbord RM, Egger M, Sterne JA (2006). A modified test for small-study effects in meta-analyses of controlled trials with binary endpoints. Stat Med.

[28] Higgins JP (2008). Commentary: Heterogeneity in meta-analysis should be expected and appropriately quantified. Int J Epidemiol.

[29] Acquaye JK, Tettey-Donkor D (2000). Frequency of hepatitis C virus antibodies and elevated serum alanine transaminase levels in Ghanaian blood donors. West Afr J Med.

[30] Adoba P, Boadu SK, Agbodzakey H, Somuah D, Ephraim RK, Odame EA (2015). High prevalence of hepatitis B and poor knowledge on hepatitis B and C viral infections among barbers: a cross-sectional study of the Obuasi municipality, Ghana. BMC Public Health.

[31] Adjei AA, Armah HB, Gbagbo F, Ampofo WK, Quaye IK, Hesse IF, Mensah G (2006). Prevalence of human immunodeficiency virus, hepatitis B virus, hepatitis C virus and syphilis among prison inmates and officers at Nsawam and Accra, Ghana. J Med Microbiol.

[32] Adjei AA, Armah HB, Gbagbo F, Ampofo WK, Boamah I, Adu-Gyamfi C, Asare I, Hesse IF, Mensah G (2008). Correlates of HIV, HBV, HCV and syphilis infections among prison inmates and officers in Ghana: A national multicenter study. BMC Infect Dis.

[33] Allain JP, Opare-Sem O, Sarkodie F, Rahman R, Owusu-Ofori S (2009). Deferred donor care in a regional hospital blood center in Ghana. Transfusion.

[34] Allain JP, Sarkodie F, Asenso-Mensah K, Owusu-Ofori S (2010). Relative safety of first-time volunteer and replacement donors in West Africa. Transfusion.

[35] Amidu N, Owiredu WB, Addai-Mensah O, Alhassan A, Quaye L, Batong B (2010). Seroprevalence and risk factors for human immunodeficiency virus, hepatitis B and C viruses infections among blood donors at the Bolgatanga Regional Hospital in Bolgatanga, Ghana. J Ghana Sci Assoc.

[36] Apea-Kubi KA, Yamaguchi S, Sakyi B, Ofori-Adjei D (2006). HTLV-1 and other viral sexually transmitted infections in antenatal and gynaecological patients in Ghana. West Afr J Med.

[37] Blankson A, Wiredu EK, Adjei A, Tettey Y (2005). Seroprevalence of hepatitis B and C viruses in cirrhosis of the liver in Accra, Ghana. Ghana Med J.

[38] Candotti D, Sarkodie F, Allain JP (2001). Residual risk of transfusion in Ghana. Br J Haematol.

[39] Candotti D, Temple J, Sarkodie F, Allain JP (2003). Frequent recovery and broad genotype 2 diversity characterize hepatitis C virus infection in Ghana, West Africa. J Virol.

[40] Ephraim R, Nsiah P, Osakunor D, Adoba P, Sakyi S, Anto E (2014). Seroprevalence of hepatitis B and C viral infections among type 2 diabetics: a cross-sectional study in the cape coast metropolis. Ann Med Health Sci Res.

[41] Ephraim R, Donkor I, Sakyi SA, Ampong J, Agbodjakey H (2015). Seroprevalence and risk factors of hepatitis B and hepatitis C infections among pregnant women in the Asante Akim North Municipality of the Ashanti region, Ghana; a cross sectional study. Africa Health Sci.

[42] King S, Adjei-Asante K, Appiah L, Adinku D, Beloukas A, Atkins M, Sarfo SF, Chadwick D, Phillips RO, Geretti AM (2015). Antibody screening tests variably overestimate the prevalence of hepatitis C virus infection among HIV-infected adults in Ghana. J Viral Hepat.

[43] Kubio C, Tierney G, Quaye T, Nabilisi JW, Ziemah C, Zagbeeb SM, Shaw S, Murphy WG (2012). Blood transfusion practice in a rural hospital in Northern Ghana, Damongo, West Gonja District. Transfusion.

[44] Lassey AT, Damale NK, Bekoe V, Klufio CA (2004). Hepatitis C virus seroprevalence among mothers delivering at the Korle-Bu Teaching Hospital, Ghana. East Afr Med J.

[45] Martinson FE, Weigle KA, Mushahwar IK, Weber DJ, Royce R, Lemon SM (1996). Seroepidemiological survey of hepatitis B and C virus infections in Ghanaian children. J Med Virol.

[46] Nkrumah B, Owusu M, Frempong HO, Averu P (2011). Hepatitis B and C viral infections among blood donors from rural Ghana. Ghana Med J.

[47] Owusu-Ofori S, Temple J, Sarkodie F, Anokwa M, Candotti D, Allain JP (2005). Predonation screening of blood donors with rapid tests: implementation and efficacy of a novel approach to blood safety in resource-poor settings. Transfusion.

[48] Sagoe KW, Agyei AA, Ziga F, Lartey M, Adiku TK, Seshi M, Arens MQ, Mingle JA (2012). Prevalence and impact of hepatitis B and C virus co-infections in antiretroviral treatment naïve patients with HIV infection at a major treatment center in Ghana. J Med Virol.

[49] Sarkodie F, Adarkwa M, Adu-Sarkodie Y, Candotti D, Acheampong JW (2001). Screening for viral markers in volunteer and replacement blood donors in West Africa. Vox Sang.

[50] Walana W, Ahiaba S, Hokey P, Vicar EK, Acuqah SEK, Der EM, Ziem TB (2014). Sero-prevalence of HIV, HBV and HCV among blood donors in the Kintampo Municipal Hospital, Ghana. British Microbiol Res J.

[51] Wansbrough-Jones MH, Frimpong E, Cant B, Harris K, Evans MR, Teo CG (1998). Prevalence and genotype of hepatitis C virus infection in pregnant women and blood donors in Ghana. Trans R Soc Trop Med Hyg.

[52] Holtzman D. Hepatitis C. http://wwwnc.cdc.gov/travel/yellowbook/2016/infectious-diseases-related-to-travel/hepatitis-c. Accessed 02 Mar 2016.

[53] Daw MA. Transmission of Hepatitis C. http://www.esciencecentral.org/ebooks/hepatitis/transmission-of-hepatitis-c-virus.php. Accessed 05 June 2016.

[54] Sandesh K, Varghese T, Harikumar R, Beena P, Sasidharan VP, Bindu CS, Tony J, Harish K, Sunilkumar K, Ramachandran TM (2006). Prevalence of hepatitis B and C in the normal population and high risk groups in north Kerala. Trop Gastroenterol.

[55] Wang CS, Chang TT, Yao WJ, Chou P (2002). Comparison of hepatitis B virus and hepatitis C virus prevalence and risk factors in a community-based study. Am J Trop Med Hyg.

[56] Edgren G, Tran TN, Hjalgrim H, Rostgaard K, Shanwell A, Titlestad K, Wikman A, Norda R, Jersild C, Wideroff L, Gridley G, Adami J, Melbye M, Nyrén O, Reilly M (2007). Improving health profile of blood donors as a consequence of transfusion safety efforts. Transfusion.

[57] Ofori-Asenso R, Agyeman A (2016). Hepatitis B in Ghana: a systematic review and meta-analysis of prevalence studies (1995–2015). BMC Infect Dis.

[58] World Health Organization. Ghana National blood Policy. http://www.who.int/bloodsafety/transfusion_services/GhanaNationalBloodPolicy2006.pdf. Accessed 10 Jan 2016.

[59] Yeung CY, Lee HC, Chan WT (2014). Vertical transmission of hepatitis C virus: current knowledge and perspectives. World J Hepatol.

[60] Reddick KL, Jhaveri R, Gandhi M, James AH, Swamy GK (2011). Pregnancy outcomes associated with viral hepatitis. J Viral Hepat.

[61] Pergam SA, Wang CC, Gardella CM, Sandison TG, Phipps WT, Hawes SE (2008). Pregnancy complications associated with hepatitis C: data from a 2003–2005 Washington state birth cohort. Am J Obstet Gynecol.

[62] Mutocheluh M, Kwarteng K (2015). Knowledge and occupational hazards of barbers in the transmission of hepatitis B and C was low in Kumasi, Ghana. Pan Afr Med J.

[63] Cheng A, Jose J, Larsen-Reindorf R, Small C, Nde H, Dugas L, Ehrhardt S, Nelson K, Ezeanolue E, Layden J (2015). A survey study of pregnant women and healthcare practitioners assessing the knowledge of attitudes and practices of hepatitis B management at a teaching Hospital in Kumasi, Ghana, West Africa. Open Forum Infect Dis.

[64] World Health Organization. Hepatitis C. http://www.who.int/mediacentre/factsheets/fs164/en/. Accessed 08 Mar 2016.

[65] Trading economics. Improved water source (% of population with access) in Ghana http://www.tradingeconomics.com/ghana/improved-water-source-percent-of-population-with-access-wb-data.html. Accessed 08 Mar 2016.

[66] World Bank. Poverty reduction in Ghana: Progress and Challenges http://www.worldbank.org/en/country/ghana/publication/poverty-reduction-ghana-progress-challenges. Accessed 02 Mar 2016.

[67] Dennis-Antwi J, Adjei S, Asare JB, Twene R. A national Survey on prevalence and social consequences of substance (drug) use among second cycle and out of school youth in Ghana. http://www.who.int/countries/gha/publications/substance_abuse_report.pdf. Accessed June 5 2016.

[68] Gokhale K, Kitamura M. $10 Copy of Gilead Blockbuster Sovaldi Appears in Bangladesh. http://www.bloomberg.com/news/articles/2015-03-08/-10-copy-of-gilead-s-blockbuster-sovaldi-appears-in-bangladesh. Accessed 02 Mar 2016.

[69] World Health Organization. Guidelines for the screening, care and treatment of persons with hepatitis C infection http://www.who.int/hiv/pub/hepatitis/hepatitis-c-guidelines/en/. Accessed Mar 15 2016. 27227200

[70] Lemoine M, Eholié S, Lacombe K (2015). Reducing the neglected burden of viral hepatitis in Africa: strategies for a global approach. J Hepatol.

[71] Alter MJ, Kuhnert WL, Finelli FL. Guidelines for laboratory testing and result reporting of antibody to hepatitis C virus*. http://www.cdc.gov/mmwr/preview/mmwrhtml/rr5203a1.htm. Accessed 08 Mar 2016. 12585742

[72] Fox RK. Core concepts. Diagnosis of acute HCV infection. http://www.hepatitisc.uw.edu/go/screening-diagnosis/acute-diagnosis/core-concept/all. Accessed 08 Mar 2016

[73] Ghana Statistical Service. 2010 Population and Housing Census; Summary report of final results.http://www.statsghana.gov.gh/docfiles/2010phc/Census2010_Summary_report_of_final_results.pdf. Accessed 01 Mar 2016.

[74] Gelberg L, Robertson MJ, Arangua L, Leake BD, Sumner G, Moe A, Andersen RM, Morgenstern H, Nyamathi A (2012). Prevalence, distribution, and correlates of hepatitis C virus infection among homeless adults in Los Angeles. Public Health Rep.

